# Quasi-Static Current Measurement with Field-Modulated Spin-Valve GMR Sensors †

**DOI:** 10.3390/s19081882

**Published:** 2019-04-20

**Authors:** Jen-Tzong Jeng, Xuan-Thang Trinh, Chih-Hsien Hung, Chih-Cheng Lu

**Affiliations:** 1 Department of Mechanical Engineering, National Kaohsiung University of Science and Technology, Kaohsiung 80778, Taiwan; jtjeng@nkust.edu.tw; 2 Faculty of Mechanical Engineering, Hung Yen University of Technology and Education, Hung Yen 160000, Vietnam; xuanthangcdt@gmail.com; 3 Graduate Institute of Manufacturing Technology, National Taipei University of Technology, Taipei 10608, Taiwan; tommy071129@gmail.com; 4 Department of Intelligent Automation Engineering, National Taipei University of Technology, Taipei 10608, Taiwan

**Keywords:** giant magnetoresistance (GMR), spin-valve, current measurement, AC modulation

## Abstract

A non-contact current measurement device comprised of a GMR sensor and a ferrite ring core was investigated. The sensor chip employed a high-sensitivity spin-valve full-bridge GMR sensor of which the direct output has non-negligible hysteresis and a limited linear range. By applying an AC modulation current to modulate the output of the GMR sensor, the hysteresis was reduced, and the linear range was over ±0.5 A. The resolution for DC and quasi-static current measurement was 0.1 mA at a 10 Hz bandwidth. The output in proportion to the measured current was obtained either by demodulating the current-sensitive AC signal or by employing the filtered output of the intrinsically nonlinear spin-valve response. The proposed current sensing scheme is suitable for quasi-static current measurement from DC to over 100 Hz.

## 1. Introduction

Magnetic field sensors or magnetometers are a class of physical devices that are able to detect and measure both field magnitude and direction simultaneously. There is a variety of sensors available in the category, such as Hall sensors, fluxgates or micro-fluxgates, anisotropic magnetoresistance (AMR), giant magnetoresistance (GMR), tunneling magnetoresistance (TMR), and giant magnetoimpedance (GMI) sensors. Of these devices, due to the micro-level dimension, excellent sensitivity, and CMOS compatibility, GMR sensors have attracted more consideration and interest for conventional applications such as detection of weak magnetic fields, angular velocity and acceleration, and inertia navigation and positioning; also for recently emerging applications like ultra-low pressure measurement [1], biological magnetic signals for gene expression analysis [2] and influenza virus detection [3], some novel technologies in water pollution detection [4] and wireless charging for electric vehicles (EVs) [5], and even current monitoring [6] for smart power grids, etc. 

Giant magnetoresistance (GMR) sensors are promising for applications in isolated or non-contact current measurement of power electronics [7]. In order to maximize the response and minimize the footprint of the current sensor, the prevailing design is to arrange the current path, e.g., the bus bar, in the vicinity of the sensing elements. Since the interference from the environmental magnetic fields could generate a spurious output and deteriorate the noise level, the conventional layout for this type of current sensor is to integrate the full Wheatstone bridge of four GMR elements on opposite branches of a bus bar [8]. However, the integration of GMR elements in a bus bar design makes it vulnerable to the heat generated by a high injection current. In addition, the lack of symmetry in the arrangement of sensors and the current path reduces the bandwidth of sensing due to the change in the surface current distribution at high frequencies, i.e., the skin effect [9]. In contrast, the GMR current sensor with a magnetic ring shows a lower temperature coefficient, as well as a better immunity from the skin effect [10]. The bias mode is also important for enhancing the output characteristic of the GMR sensor. It has been shown that the hysteresis can be effectively reduced using the field modulation and demodulation technique [11]. The driving method with AC voltage bias and lock-in detection is also available to reduce the noise level [12], but the hysteresis remains; hence, the output offset is susceptible to an undesirable high current pulse during the measurement operation.

In this article, a non-contact current measurement device comprised of a commercial spin-valve GMR sensor and a ferrite ring core is presented and investigated. The linear output with negligible hysteresis is achieved by applying the field modulation technique. The characteristics of device sensitivities and current noise levels are measured at various modulation frequencies and modulation currents. Finally, the factors affecting the output performance are analyzed and discussed.

## 2. Methods and Experiments

### 2.1. Design of the GMR-Based Device

**Figure 1:**
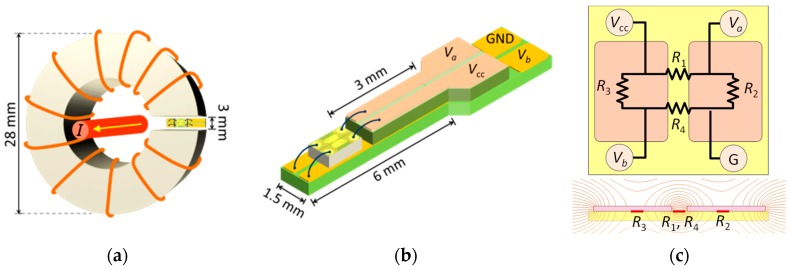
Schematics of the current sensor: (**a**) GMR sensor with a C-shaped ring-core made of ferrite; (**b**) wiring of the spin-valve chip mounted on assembled printed-circuit boards; (**c**) schematic layout of a spin-valve GMR bridge sensor with a flux concentrator.

To achieve non-contact measurement operation, the measurement device was combined with an annular magnetic ring to allow the wire to pass through the center of it. When the to-be-measured current passes, the magnetic fields generated by the current will be merged into the annular magnetic ring. The GMR sensor placed at the gap of the ring thus detects the magnetic field and results in magnetoresistance change, fulfilling the non-contact current measurement. The design of the current measurement device is shown in Figure 1. It consisted of a GMR spin-valve chip in the 3-mm gap of a C-shaped ferrite ring, which was 28 mm in outer diameter, 16 mm in inner diameter, and 13 mm in thickness. The sensor chip was a commercially-available spin-valve GMR sensor (bare die), Model GF708, from Sensitec GmbH (Lahnau, Germany). Figure 1c shows the schematic layout of the sensor chip, which consisted of four GMR elements and a pair of thin-film flux concentrators. Two of the four GMR elements were covered with magnetic shielding films to serve as reference resistors. The two sensing resistors were placed in the gap between the two magnetic shielding films, where the magnetic flux density was enhanced by one order of magnitude. The contact pads were bonded to the four terminals on assembled printed-circuit boards. According to the product datasheet, the GMR sensor had a linear response centered at about 2 Oe, and the output hysteresis was less than 1 Oe [13]. To overcome the remaining hysteresis and to shift the operation point for linear bipolar sensing, a 50-turn modulation coil was wound around the C-shaped ferrite core to generate the AC modulation signal.

### 2.2. Sensing Principles

**Figure 2:**
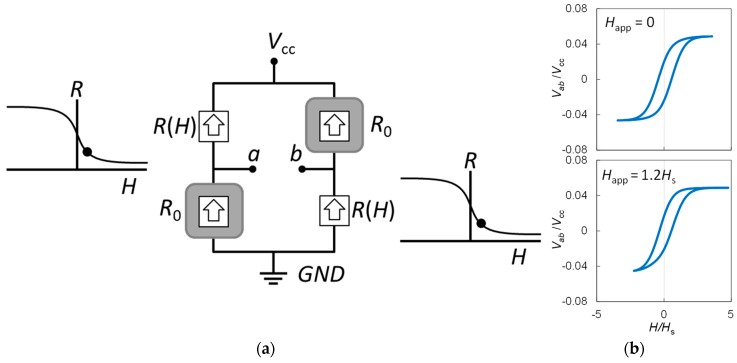
(**a**) Equivalent circuit of a spin-valve GMR bridge sensor with two shielded resistors for reference. (**b**) Voltage-field (*V*-*H*) curves of a GMR sensor simulated by using Equations (1) and (2) with and without an applied magnetic field.

The equivalent circuit of the spin-valve GMR sensor is shown in Figure 2a. The resistance of sensing elements as a function of external field is *R*(*H*), and the resistance of reference elements is fixed at *R*_0_. When an external field (*H*) is applied along the pinning direction of the sensing elements, the resistance of sensing elements decreases, which is indicated by the solid dot in the *R-H* curves in Figure 2a. The response of an ideal spin-valve GMR element to the applied field can be simulated with the following empirical formula [14]:

(1) $$R(H)=R_{0}\left(1+\frac{r}{r+2}\cdot \tanh(-\frac{H_{\mathrm{eff}}}{H_{s}})\right)$$

where *r* is the magnetoresistance ratio, *H* is the external field, *H*_eff_ = *H* + *H*_in_ ± *H*_c_ is the effective field for positive and negative sweep, *H*_in_ is the shift in operation point due to the unbalanced coupling between magnetic layers, *H*_s_ is the nominal saturation field, *H*_c_ is the coercivity, and *R*_0_ is the resistance at zero effective field. The reduction in the resistance of sensing element results in an increase in the output of GMR bridge, which can be simulated by the following formula derived from (1):

(2) $$V_{\mathrm{ab}}(H)=V_{cc}\left[\frac{r\tanh(H_{\mathrm{eff}}/H_{s})}{2(r+2)-r\tanh(H_{\mathrm{eff}}/H_{s})}\right],$$

**Figure 3:**
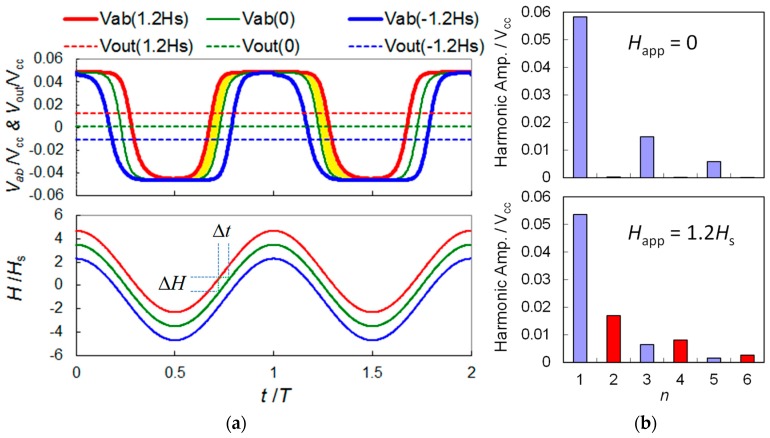
Analytic model for the modulated output of a GMR spin-valve bridge sensor: (**a**) Time trace of voltage output (*V*_ab_) and DC level (*V*_out_). The excitation waveform is also shown at the bottom for comparison, where *t* is the time and *T* denotes the cycle time. (**b**) Harmonic spectrum of *V*_ab_ with and without an applied field.

The functions *R*(*H*) and *V*_ab_(*H*) are generally non-linear and hysteretic when *H*_c_ is non-zero, and *V*_cc_ denotes DC bias voltage of the GMR bridge. An example of the simulated *R*(*H*) curve using Equation (1) fit to the experimental data is available in [14], and the typically simulated *V*_ab_(*H*) curves using Equation (2) at zero and non-zero applied field are given in Figure 2b. The parameters used are: *H*_app_ = 0 and 1.2*H*_s_, *H*_mod_ = 3.5*H*_s_, *H*_c_ = 0.5*H*_s_, *H*_in_ = −0.065*H*_s_, and *r* = 10%. It was assumed that *V*_ab_ is not a function of modulation frequency. The nonlinearity and remaining hysteresis can be eliminated by applying an AC modulation field to modulate the output of the GMR bridge sensor. The simulated time trace (*V*_ab_) and DC level output (*V*_out_) of the GMR bridge under a modulation field of *H*_exc_ = *H*_mod_ sin(ω*t*) is shown in Figure 3, where *H*_mod_ denotes the amplitude of modulation field, ω = 2π/*T* is the angular frequency, *t* is the time, and *T* is the period of the excitation waveform. The maximum time rate of change of the modulation field is in proportion to the modulation amplitude:

(3) $$\frac{dH}{dt}(t=0)=\omega H_{\mathrm{mod}}$$

where ω=2πf is the angular modulation frequency, f=1/T is the modulation frequency, and T is the cycle time. When an external field of *H*_app_ induced by the test current is applied, the total external field is *H* = *H*_app_ + *H*_exc_, which results in a shift in the *V-H* curve shown in Figure 2b. For a small external field *H*_app_ = Δ*H*, the zero-crossing points of the time trace are shifted in time by:

(4) $$\Delta t=\pm \Delta H/(dH/dt)=\pm \Delta H/\omega H_{\mathrm{mod}},$$

Equation (4) is valid when Δ*H* ≪ *H*_s_. In this case, the applied field induces a shift in the *V-H* curve by Δ*H*, as shown in Figure 2b, while the linear *H*-*t* relation is shifted by Δ*t*, as shown in the bottom of Figure 3a. As previously mentioned, the change in the DC level of *V*_ab_ due to Δ*H* can be calculated as *V*_out_:

(5) $$V_{out}\left(\Delta H\right)=\frac{1}{T}\int_{0}^{T}V_{ab}\left(H\left(t\right)\right)dt\approx \;V_{0}+\frac{1}{T}\left(4V_{max}\Delta t\right),$$

where *V*_0_ is the zero-field level and *V*_max_ = *V*_ab_(∞) is the saturation voltage of a spin-valve bridge. The averaged output levels under different external DC fields are shown as the horizontal dashed lines in Figure 3. With a positive applied field of *H*_app_ = 2*H*_s_, the time duration that *V*_ab_ stays at the maximum level (*V*_max_) becomes longer, resulting in an elevated average DC level (*V*_out_). With a negative applied field of *H*_app_ = −0.2, the *V*_out_ becomes negative. The simulation results imply that *V*_out_ has a bipolar response to the applied field. The change in output level is determined by the last term in (5), where 4*V*_max_Δ*t* corresponds to the approximate area of the shaded regions of the *V*-*t* plot in Figure 3. The harmonic spectrum with and without an applied field is given in Figure 3b, which indicates that the second harmonic has a maximum sensitivity to the applied field *H*_app_. 

**Figure 4:**
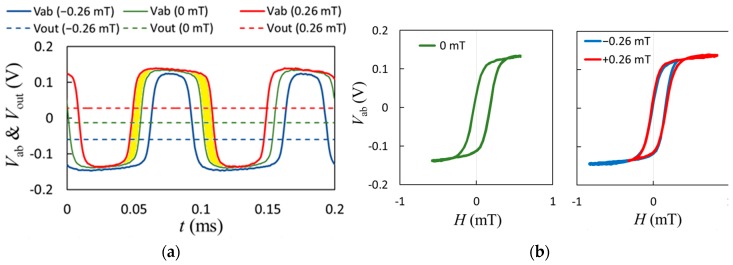
(**a**) Observed time traces of a GMR sensor; (**b**) *V-H* curves of the direct output for a GMR sensor.

The experimentally-observed time trace for the directed output of the GMR spin-valve sensor is shown in Figure 4. It was found that the product of 4*V*_max_Δ*T* approximates the yellow-shaded areas, similar to the simulation results. From (5), the average output voltage in a small-signal mode is found to be proportional to the applied field Δ*H* as follows:

(6) $$\Delta V=\frac{1}{T}(4V_{\max}\Delta t)=\frac{2V_{\max}\Delta H}{\pi H_{\mathrm{mod}}}$$

Therefore, the sensitivity to the external field for a bridge sensor can be estimated as follows:

(7) $$\frac{dV}{dH}\approx \frac{\Delta V}{\Delta H}=\frac{2V_{\max}}{\pi H_{\mathrm{mod}}}$$

The field-sensitive DC output of the sensor predicted from (7) can be obtained by simply applying a low pass filter, e.g., a first order R-C filter with a cutoff frequency at 100 Hz [15], or by employing the synchronous detection technique [11] to detect the second-harmonic response. With synchronous detection, an additional gain factor is necessary to obtain the demodulated sensitivity for the GMR sensor. 

For the current sensor in Figure 1a, the magnetic field in the air gap of the C-shaped core generated by the current passing through the *N*-turn coil wound on the core can be estimated by:

(8) $$H=\frac{NI}{l_{g}+(l_{c}/\mu_{r})},$$

where *μ*_r_ is the relative permeability, *l*_c_ is the path length of magnetic circuit in the core, and *l*_g_ is the air gap length. Given *μ*_r_ ≈ 10^3^, *l*_c_ ≈ 100 mm, and *l*_g_ = 3 mm, Equation (8) reduces to *H* ≈ *NI*/*l*_g_. This relation applies to the modulation field (*H*_exc_), as well as the external field (*H*_app_) induced by the current *I* to be detected by the GMR sensor. For *H*_exc_, *N* = 50 is the number of turns of the excitation coil, and hence, *H* ≈ 200*I*, in units of Oe/A. For *H*_app_, the current under test passes through the long conductor at the center of the C-shaped core, and hence, *N* = 1, so the relation becomes *H* ≈ 4*I* in units of Oe/A.

### 2.3. Measurement Experiment

**Figure 5:**
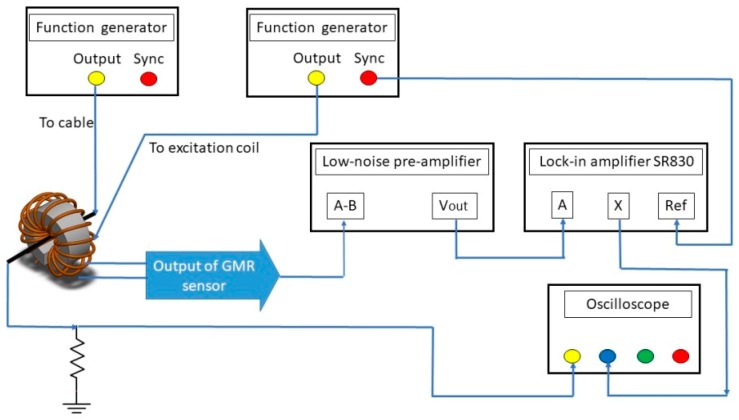
Experimental installation schematic of the GMR-based current measurement system.

To characterize the performance of the current measurement device, the experimental installation is set and shown in Figure 5. A function generator was employed to input DC or AC current to the cable through the C-shaped ring, and the other provided for a sine-wave signal to generate excitation magnetic fields. The output of the GMR sensor was connected to a pre-amplifier to amplify the sensing voltage with respect to resistance variation. The amplified signal was then filtered and processed to obtain the second-harmonic response by a lock-in amplifier SR 830. The gain was 10 for the preamplifier and 20 for the analog output of the lock-in amplifier, giving a total gain of 200 for the system. The cutoff frequency of the second-order low-pass filter in the preamplifier was 300 kHz. To test the *V*-*I* curves, the test current was swept at 0.2 Hz, and the filter bandwidth of the lock-in amplifier was 16 Hz. An oscilloscope and a DAQ board were employed to verify and record the amplitude signals from the sensor.

## 3. Results and Discussions

### 3.1. Demodulated Sensitivity

**Figure 6:**
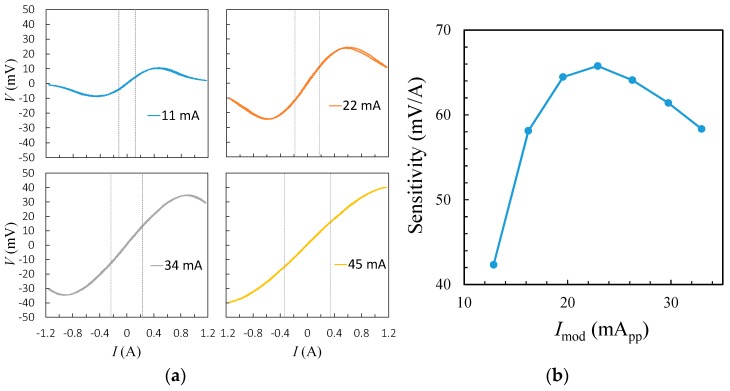
(**a**) Transfer curves of the GMR current sensor at modulation currents of 11, 22, 34, and 45 mA. The vertical dashed lines indicate the range of maximum nonlinearity less than 2%. (**b**) The sensitivities with respect to various modulation currents.

The observed sensitivities of the GMR current sensor demodulated at the second harmonic frequency under various modulation currents at 1 kHz are shown in Figure 6. The transfer schematics in Figure 6a show the sensitivities determined by sweeping the test current within the ±0.1 A range at 0.2 Hz, where the nonlinearity was better than 2%. In the open-loop mode, it was found that the linear operation range was boosted from ±0.1 A to ±0.5 A when the modulation current was increased from 11 mA to 45 mA. As the measured current-to-field transfer coefficient of the C-shaped ring coil was 14.5 mT/A, the modulation currents corresponded to the modulation fields ranging from 0.16 mT to 0.65 mT. Further increase in modulation current can expand 2% of the linear range over 1 A at the expense of a reduced sensitivity. At the modulation frequency of 1 kHz, the sensitivity was maximized to 66 mV/A under a current amplitude of 22 mA, as shown in the inset of Figure 6. At this driving condition, the hysteresis was found to be less than 1% for a complete sweep in the linear range of ±0.2 A. 

### 3.2. Frequency Dependence

**Figure 7:**
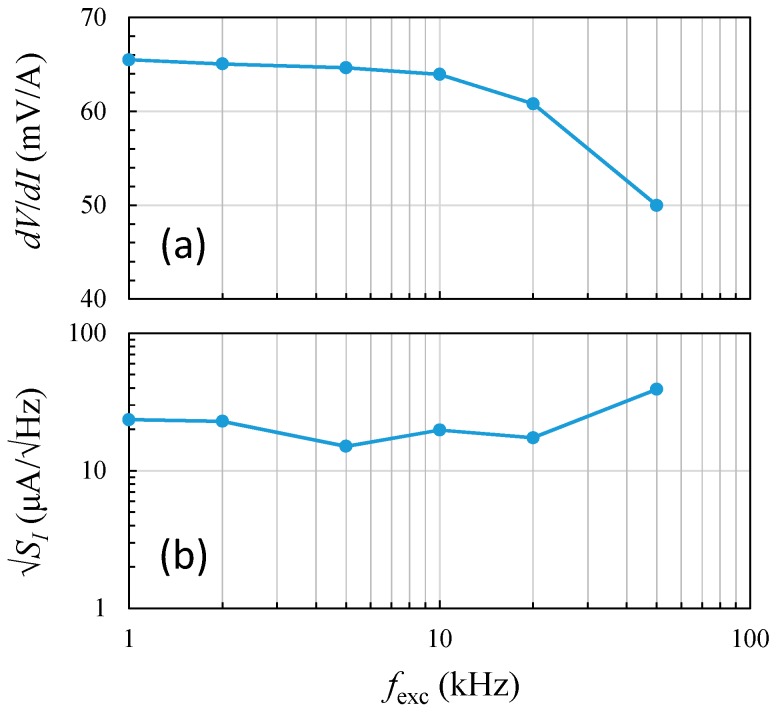
Dependence of the modulation frequency on (**a**) the maximum sensitivity and (**b**) 1-Hz current noise.

The dependence of modulation frequency on sensitivity and 1-Hz current noise from 1 kHz to 50 kHz for the GMR current sensor is shown in Figure 7. The measurement experiment was carried out using 200 mA of measured current through the ring. It can be seen that the sensitivity slightly decreased with increasing modulation frequencies from 1 kHz to 10 kHz, as shown in Figure 7a. When the modulation frequency was above 10 kHz, the sensitivity was significantly reduced. The drop in sensitivity was found to be dominated by the change in the intrinsic sensitivity of the GMR sensor when the driving frequencies were close to 100 kHz. 

In addition, it is of interest to explore the current noise level of the sensor as the signal-to-noise ratio (SNR) is relatively critical to a miniature system. Similarly, Figure 7b shows the 1-Hz current noise densities at various modulation frequencies from 1 kHz to 50 kHz, while the corresponding sensitivities are given in Figure 7a. It was found that the noise level was minimized to less than 20 μA/√Hz@1 Hz for the modulation frequencies between 5 kHz and 20 kHz. Nevertheless, the noise density gradually rose when the modulation frequency rose past 20 kHz, and the noise level reached its maximum of 39 μA/√Hz@1 Hz at a 50-kHz modulation. Noticeably, the excitation frequency dependence on sensitivity and noise was relatively opposite, particularly at higher modulation frequencies. The increase in current noise was mainly due to the reduced sensitivity of the GMR sensor, which reached 50 mV/A at a higher frequency of 50 kHz. Hence, the trade-off between sensitivity and noise density was critical and feasible.

As the bandwidth of a modulated GMR sensor must be less than the limit set by the characteristic frequency of the low-pass filter in the demodulation circuit, the modulation frequency should be as high as possible in order to achieve the maximum available bandwidth. For the 50-kHz modulation, the filter bandwidth would be on the order of 1 kHz, which sets the upper limit of the bandwidth for a modulated GMR current sensor. Nevertheless, it is still possible to implement a high-bandwidth current measurement system by employing the combined low-frequency and high-frequency GMR sensors [15].

**Figure 8:**
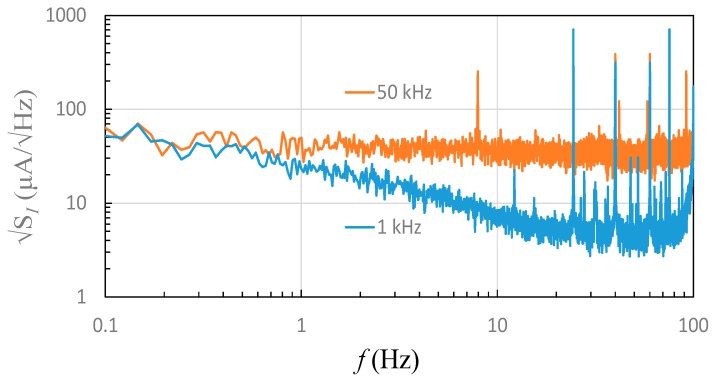
Current noise spectra under 1-kHz and 50-kHz modulation fields under the maximum sensitivity condition.

On the performance of current noise, the current noise spectra of the GMR sensor at 1 kHz and 50 kHz modulation frequency respectively were investigated, as shown in Figure 8. With a 1-kHz modulation current of 22 mA, i.e., under the maximum sensitivity condition, the 1-Hz noise was found to be 24 μA/√Hz, while the 1-Hz noise at a 50-kHz modulation current was almost constant around the average level of 33 μA/√Hz. Furthermore, one can see that the low frequency 0.1-Hz noise was about 66 μA/√Hz, which was similar to the value with 1-kHz modulation. Yet, when current frequency increased, the noise level with 1-kHz modulation was expected to decrease, but the other with 50-kHz modulation remained nearly flat and constant. The corresponding root-mean-squared noises were 76 μA and 104 μA, respectively, for 1-kHz and 50-kHz modulations when a bandwidth of 10 Hz was assumed. The nearly flat noise spectrum with 50-kHz modulation indicates that the proposed current sensing system is capable of operation from DC to over 100 Hz with a moderate noise level.

## 4. Conclusions

A non-contact current technique based on the combination of a GMR spin-valve sensor and a C-shaped ferrite ring core was investigated. The linear range was ±0.5 A with negligible hysteresis, and the resolution for DC and quasistatic current measurement was 0.1 mA at a 10-Hz bandwidth. The bandwidth can be further expanded by combining with a high-frequency current sensor. The GMR-based current measurement techniques are considered promising for various applications including non-contact current sensing for wireless battery charging, non-interrupting circuit probes, and long-term current monitoring for smart power grids.
